# Revisiting T-cell adhesion molecules as potential targets for cancer immunotherapy: CD226 and CD2

**DOI:** 10.1038/s12276-024-01317-9

**Published:** 2024-10-01

**Authors:** Yunju Jo, Hye-In Sim, Bohwan Yun, Yoon Park, Hyung-seung Jin

**Affiliations:** 1 Chemical and Biological Integrative Research Center, Biomedical Research Institute, Korea Institute of Science and Technology (KIST), Seoul, South Korea; 2 Department of Convergence Medicine, Asan Institute for Life Sciences, Asan Medical Center, University of Ulsan College of Medicine, Seoul, South Korea

**Keywords:** Tumour immunology, Tumour biomarkers

## Abstract

Cancer immunotherapy aims to initiate or amplify immune responses that eliminate cancer cells and create immune memory to prevent relapse. Immune checkpoint inhibitors (ICIs), which target coinhibitory receptors on immune effector cells, such as CTLA-4 and PD-(L)1, have made significant strides in cancer treatment. However, they still face challenges in achieving widespread and durable responses. The effectiveness of anticancer immunity, which is determined by the interplay of coinhibitory and costimulatory signals in tumor-infiltrating immune cells, highlights the potential of costimulatory receptors as key targets for immunotherapy. This review explores our current understanding of the functions of CD2 and CD226, placing a special emphasis on their potential as novel agonist targets for cancer immunotherapy. CD2 and CD226, which are present mainly on T and NK cells, serve important functions in cell adhesion and recognition. These molecules are now recognized for their costimulatory benefits, particularly in the context of overcoming T-cell exhaustion and boosting antitumor responses. The importance of CD226, especially in anti-TIGIT therapy, along with the CD2‒CD58 axis in overcoming resistance to ICI or chimeric antigen receptor (CAR) T-cell therapies provides valuable insights into advancing beyond the current barriers of cancer immunotherapy, underscoring their promise as targets for novel agonist therapy.

## Advancing cancer immunotherapy: role of CD226 and CD2 in tumor immunity

Immunotherapy, including immune checkpoint inhibitors and adoptive T-cell therapy, has transformed cancer treatment, yet durable responses are limited to a minority of patients. Anticancer immunity, shaped by co-inhibitory and co-stimulatory signals in tumor-infiltrating immune cells, underscores co-stimulatory receptors as promising targets for immunotherapy. This review explores the roles of CD226 and CD2 in regulating T cell responses, particularly in tumor immunity. CD2 and CD226, primarily on T and NK cells, are crucial for cell adhesion and recognition, known for their role in overcoming T cell exhaustion and boosting anti-tumor responses. The review examines their potential as targets in novel cancer immunotherapeutic strategies. This summary was initially drafted using artificial intelligence, then revised and fact-checked by the author.

## Introduction

Immunotherapy has transformed cancer treatment by significantly improving patient outcomes through treatments such as immune checkpoint inhibitors (ICIs) and adoptive T-cell therapy (ACT)^1^. While immunotherapy has shown promising results across various cancer types, durable responses are seen only in a minority of patients. Several efforts, such as triggering costimulatory signals with agonistic antibodies, have continued to overcome its challenges and expand its effectiveness.

Costimulation is crucial for full T-cell activation along with cytokine support, as stimulation solely through the T-cell receptor (TCR) complex can induce anergy^2^. In particular, it is well known that tumors can impede proper T-cell priming to tumor antigens, a key factor in suppressing the antitumor response. Therapies targeting potent costimulatory signal-delivering receptors, such as 4-1BB, OX40, GITR, ICOS and CD40, have been developed to overcome this challenge. Although agonistic antibodies targeting these receptors have shown promising preclinical effects, their clinical use has been limited by their narrow therapeutic window. This limitation is primarily due to their transient expression upon stimulation, strong constitutive stimulation-induced T-cell dysfunction, and on-target/off-tumor toxicity^3–6^. Ongoing efforts are being made to overcome the existing limitations of T-cell agonist therapy, including the development of antibodies with tumor-specific activity and the induction of oligomerization in tumor necrosis factor (TNF) receptor superfamily (TNFRSF) costimulatory receptors. Combination treatments with other therapeutics, including chemotherapy or radiotherapy, are also being explored. However, challenges such as their transient expression and strong signaling-induced immune deletion still impact their clinical efficacy. Therefore, novel costimulatory targets capable of activating T cells through alternative mechanisms must be identified.

The role of T-cell adhesion costimulatory receptors in regulating tumor immunity has been relatively underexplored. Their function as adhesion molecules has been emphasized more than their costimulatory activity. However, recent studies have revealed a positive correlation between the expression of CD226 or CD2-CD58 and clinical outcomes across various tumor types in response to cancer treatment, including ICIs and chimeric antigen receptor (CAR) T-cell therapies. Both CD226 and CD2 are T-cell adhesion molecules that closely associate with the TCR upon antigen engagement^7,8^. Their role as costimulatory receptors in tumor immunity is gaining recognition, especially in mitigating T-cell exhaustion. Unlike TNFRSF costimulatory receptors, CD226 and CD2 are constitutively expressed by T cells. Recently, the importance of CD226 in tumor immunity has emerged, particularly in the context of anti-TIGIT therapy^9,10^. CD226 competes with TIGIT, a coinhibitory receptor, for ligand binding and delivers costimulatory signals to T cells, suggesting that it has critical clinical implications in anti-TIGIT therapy. The loss of CD58, a ligand of CD2, is often observed in tumors resistant to ICIs or CAR T-cell therapies, highlighting the importance of the CD2‒CD58 axis in regulating T-cell antitumor responses^11–16^. While further investigation of the roles of these molecules in tumor immunity is required, they hold promise as potential targets for novel agonist therapy. Their characteristics offer the potential to overcome the limitations of existing agonist therapies while leveraging their advantages as adhesion molecules.

In this review, we focus on the roles of CD226 and CD2 in modulating the responses of T cells, especially within the context of tumor immunity. In addition, we explored their potential as targets for novel cancer immunotherapies.

## CD226

### CD226 expression, structure, and ligands

CD226, also known as DNAX accessory molecule 1 (DNAM-1), is an immunoglobulin-like transmembrane glycoprotein consisting of two immunoglobulin V-like domains, a type-I transmembrane domain, and an intracellular domain (ICD) with an immunoglobulin tail tyrosine (ITT). CD226 is broadly expressed at varying levels across various cell types, including T cells, NK cells, NKT cells, small subsets of B cells, monocytes/macrophages, dendritic cells (DCs), megakaryocyte/platelet lineages, hematopoietic precursor cells, endothelial cells, and mast cells^17^. These findings indicate its involvement in regulating various cellular functions.

CD226 genetic polymorphisms are correlated with various immune pathologies, highlighting their pivotal role in immune regulation^18–20^. Notably, the nonsynonymous mutation CD226 rs763361/gly307ser is associated with increased susceptibility to autoimmune diseases such as type 1 diabetes, rheumatoid arthritis, multiple sclerosis, autoimmune thyroid disease, and systemic sclerosis^21–24^. Another polymorphism, CD226 rs727088, might influence CD226 expression at the transcriptional level^25^. Further investigations are needed to understand the influence of this mutation on disease occurrence and development.

**Fig. 1:**
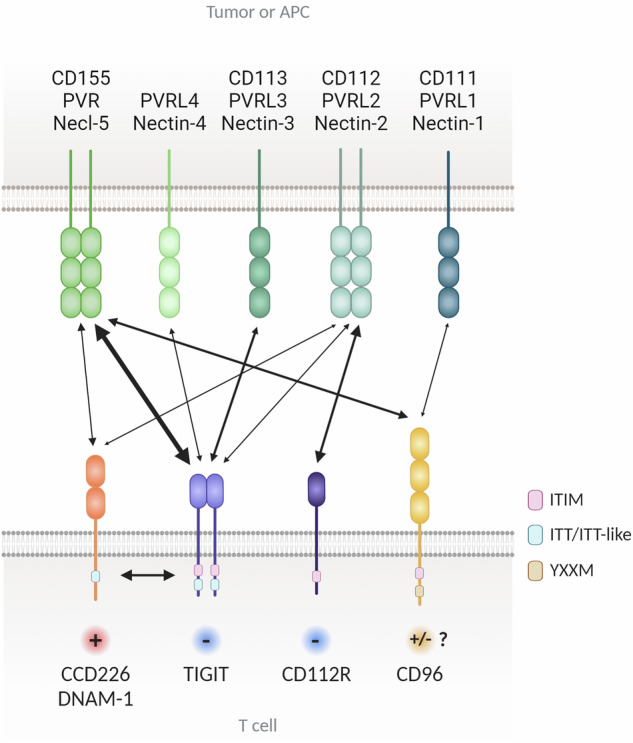
The TIGIT/CD226/CD96/CD112R axis. TIGIT, CD226, CD96, and CD112R are expressed mainly on T cells and NK cells. The ligands PVR, Nectin-1, Nectin-2, Nectin-3 and Nectin-4 are expressed on tumor cells and antigen-presenting cells (APCs). TIGIT binds PVR, Nectin-2, Nectin-3, and Nectin-4, while CD226 binds PVR and Nectin-2, and CD96 binds PVR and Nectin-1. CD226 competes with TIGIT and CD96 for PVR binding and with CD112R for Nectin-2 binding. The cytoplasmic tails of TIGIT, CD96, CD112R, and PVR contain ITIM motifs that initiate inhibitory signals, while TIGIT also features an ITT-like motif. CD226 binds PVR to transmit positive signals and associates with LFA-1. The signaling outcome of CD96 binding to CD155 in human T cells is still unclear. CD112R binds CD112 to deliver an inhibitory signal via its ITIM. Two-sided arrows indicate receptor‒ligand interactions, and their size is proportional to the reported affinities. Keywords: APCs (antigen-presenting cells), ITIM (immunoreceptor tyrosine-based inhibitory motif), ITT (Ig tail-tyrosine), NK cells (natural killer cells), TIGIT (T-cell immunoreceptor with immunoglobulin and ITIM domains).

CD226 interacts with PVR (CD155) and nectin-2 (CD112 and PVRL2) (Fig. 1)^26,27^. The extracellular domain (ECD) of CD226 features an unconventional side-by-side arrangement of the D1 and D2 domains, which seemingly collapse upon each other. The CD226/PVR interaction relies on conserved lock-and-key motifs in their D1 domains, while its collapsed architecture positions the D2 domain for direct contact^28,29^. The binding affinity measured in solution between human CD226-Fc and PVR-Fc proteins closely resembles that of CD226-Fc and nectin-2-Fc. However, CD226-Fc is less efficient at binding to nectin-2 than PVR-expressing cells are, indicating that the homophilic interaction of nectin-2 could impede the binding of CD226 to nectin-2^30^. Structural analysis indicated that PVR lacks strong homophilic interactions. In addition, while nectin-2 can function as a monomer, it also shows a tendency for homodimerization^30–32^. Both PVR and nectin-2 can modulate lymphocyte activity through interactions with various receptors. In particular, PVR interacts with CD226, TIGIT, and CD96, whereas nectin-2 binds to CD226, TIGIT, and CD112 receptor (CD112R), also known as PVRIG (Fig. 1). Like CD28 in the B7/CTLA-4 axis, CD226 has a lower affinity for its shared ligands PVR and nectin-2 than for the inhibitory receptors TIGIT, CD96, and CD112R^33^. This establishes appropriate immune regulation tailored to the surrounding environment through competitive inhibition. PVR and nectin-2 are widely expressed in a variety of tissues, including on epithelial cells, myeloid cells, and pathogen-infected cells. Toll-like receptor signaling in antigen-presenting cells (APCs) can induce the expression of PVR and nectin-2 through the NF-κB pathway^34,35^. Moreover, PVR and nectin-2 are commonly present at increased levels in various tumor cells, including both hematological and solid malignancies^36^. Increased PVR expression in tumors is correlated with decreased activity of tumor-infiltrating lymphocytes (TILs) and a poorer prognosis across various cancer types. Furthermore, PVR expression has been identified as a predictor of the response to ICIs^37,38^.

### CD226 signaling

The ICD of CD226 contains tyrosine and serine residues that are critically involved in signaling, Y322 and S329 in humans (Y319 and S326 in their murine counterparts)^8^. Upon engagement with PVR and nectin-2, protein kinase C (PKC) phosphorylates the S329 residue of CD226^39^. This results in the association of lymphocyte function-associated antigen-1 (LFA-1) with CD226, which in turn facilitates the phosphorylation of CD226 at Y322 by the Src family kinase FYN^40^. This initiates subsequent downstream signaling induced by CD226, resulting in the phosphorylation of SLP-76 and Vav1^41^. This phosphorylation activates phosphatidylinositol-4,5-bisphosphate phosphodiesterase gamma-2 (PLCγ2), leading to the activation of the extracellular signal-regulated kinase (ERK) and AKT pathways. Activated AKT phosphorylates the forkhead box protein O1 (FOXO1) transcription factor, triggering its translocation from the nucleus to the cytoplasm^42^. In the cytoplasm, FOXO1 undergoes degradation and inactivation, effectively eliminating the negative regulator of NK-cell activation. Considering the critical roles of FOXO1 in T cells, further exploration is needed to understand the impact of CD226-mediated suppression on the regulation of T-cell functions. The significance of CD226 Y319 phosphorylation has been assessed in CD226Y319F KI (knock-in) mice, revealing impaired cytotoxicity and cytokine production by NK cells^43^. A similar observation regarding CD226 phosphorylation at Y322 has been made in human T cells. Exogenous expression of CD226 wildtype (WT) or CD226 Y322A in human T cells indicates that PVR-induced CD226 phosphorylation at Y322 is essential for downstream signaling activation, including ERK, p38, and AKT, and subsequent T-cell responses^9^. In addition, the G307S mutation in CD226 has been shown to promote increased phosphorylation of Y322 and recruitment of Lck, thereby augmenting proinflammatory cytokine production in CD4 + T cells. Nonetheless, the molecular mechanism by which the G307S mutation enhances Lck recruitment to CD226 remains uncertain^44^.

#### CD226 in immunological synapse formation

Upon successful recognition of APCs by T cells, a specialized organization of membrane proteins is formed at the contact area through cytoskeletal remodeling and receptor rearrangement, termed the immunological synapse (IS). This structure immobilizes cell movement, prolonging the contact time between the two cells and facilitating numerous molecular interactions between receptors, many of which are short-lived with low affinities^45^. Upon T-cell activation, the IS reorganizes, forming central supramolecular activation complexes (cSMAC) containing the TCR complex and CD2/CD28 receptors. Adhesion molecules such as LFA-1 encircle this core, defining the peripheral SMAC (pSMAC), which is then surrounded by the distal SMAC (dSMAC). Initially, excluded from TCR-pMHC microclusters during IS formation, CD45 molecules migrate to the dSMAC, where the corolla structure begins to form^46,47^.

CD226 is known to promote the formation of IS by recruiting actin-binding proteins such as Discs-large and 4.1 G and facilitating the aggregation of LFA-1^48–51^. Upon T-cell activation with anti-CD3, anti-CD28, or anti-CD18 monoclonal antibodies (mAbs), CD226 is directed to lipid rafts through serine phosphorylation-mediated binding to LFA-1 and initiating LFA-1-mediated costimulatory signaling via phosphorylation at Y322 of CD226^40,52,53^. Domain mapping and structural studies have revealed the ligand binding-dependent role of CD226 in IS formation. These studies demonstrated that the D1 domain-mediated binding of CD226 to PVR and nectin-2 enhances NK cell cytotoxicity against target cells by facilitating cell-to-cell conjugation and IS formation^28,29,54^. Furthermore, a recent study using nanoscopic imaging techniques revealed that CD226 in activated human T cells accumulates at the IS upon incubation with planar lipid bilayers (PLBs) containing PVR but not nectin-1 (CD111 and PVRL1)^55^.

Studies with CD226-deficient T cells further emphasize the role of CD226 in optimal IS formation. When CD226-deficient naïve T cells are stimulated with peptide-pulsed DCs, they display normal polarity, such as actin and LFA-1 polarization and localization of the microtubule organizing center (MTOC) to the uropod, but fail to form conjugates with DCs, resulting in reduced expansion^56^. However, normal activation of CD226-deficient naïve T cells was observed upon CD3/CD28 bead stimulation, indicating that CD226 primarily plays a role in cell‒cell conjugation during the T-cell priming process. When the cytotoxicity of preactivated CD226-deficient OT-I T cells against MC38-OVA tumor cells was assessed, decreased tumor cell killing was observed due to the suboptimal formation of the IS. Another study reported the non-redundant role of CD226 costimulation in conventional CD8 + T cells interacting with non-professional APCs such as tumor cells but not DCs^57^. This discrepancy in the requirement for CD226 in DC-mediated T-cell activation may be caused by differences in sensitivity to antigens between naïve and conventional T cells. Just as tumor cells evade recognition by other costimulatory molecules, leading to reduced T-cell antigen sensitivity, naïve T cells may require additional costimulation by CD226 to increase their sensitivity to antigen recognition.

#### Mechanisms regulating CD226 activity

##### Extracellular regulation

**Fig. 2:**
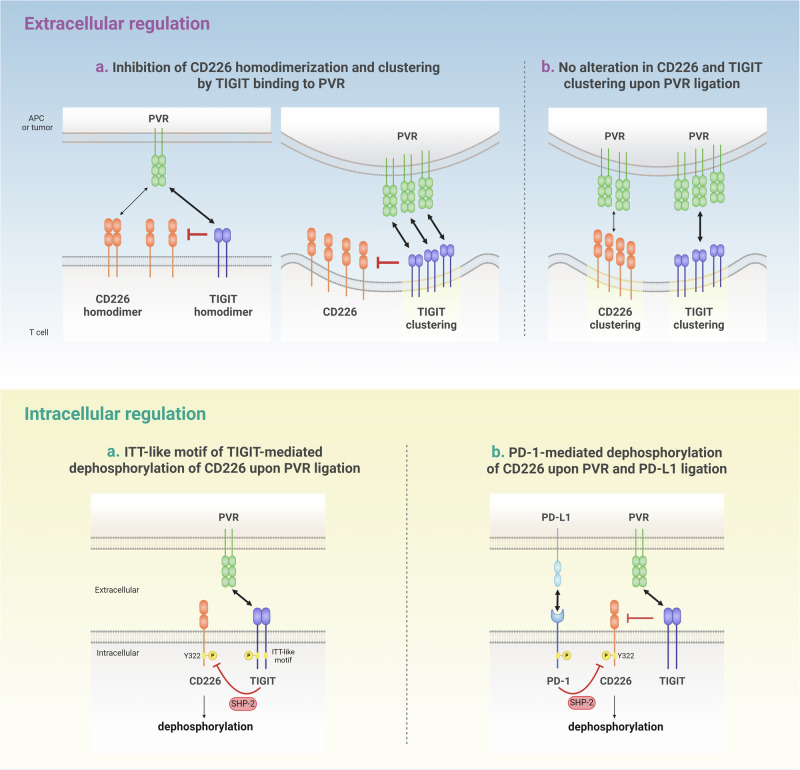
Regulation of CD226 activity: extracellular and intracellular mechanisms. CD226 activity is regulated through two main mechanisms: competitive ligand binding at the extracellular level and SHP-2-mediated tyrosine dephosphorylation at the intracellular level. With respect to the extracellular regulation of CD226, there are two main hypotheses: a. TIGIT binds to PVR with a higher affinity than does CD226 and inhibits the homodimerization of CD226. This inhibition leads to a decreased accumulation of CD226 at the sites of cell-to-cell contact. b. In addition to ligand engagement, CD226 and TIGIT can colocalize at the IS, and the presence of CD226 does not affect TIGIT clustering. In terms of intracellular regulation, there are two proposed mechanisms for regulating the tyrosine phosphorylation of CD226: a. The binding of TIGIT to PVR, which induces the phosphorylation of the ITT-like motif of TIGIT, recruits SHP-2. This results in the dephosphorylation of tyrosine 322 on CD226. b. TIGIT prevents the interaction between CD226 and PVR through its ECD, but its ICD does not influence the phosphorylation of CD226. Instead, PD-1 recruits SHP-2, which leads to the dephosphorylation of CD226.

CD226 activation can be inhibited by its counterpart TIGIT, which outcompetes CD226 for binding to PVR or nectin-2 with a higher affinity than CD226^58,59^. Studies employing time-resolved FRET (TR-FRET) suggest that TIGIT interferes with CD226 homodimerization in an ECD-dependent manner^10^. A previous structural study reported the *cis*-homodimerization of TIGIT, facilitating the conjugation of TIGIT-expressing cells to PVR-expressing cells^60^ (Fig. 2). However, a recent study by Worboys et al. demonstrated that TIGIT clustering at the IS remained intact even with an inert mutation in its homodimerization site (I42D)^55^. Nevertheless, it remains unclear whether CD226 also undergoes structural homodimerization. More importantly, further investigation is needed to determine whether the homodimerization of TIGIT or CD226 is induced by ligand binding or if it occurs constitutively. Another mechanism of TIGIT-mediated CD226 inhibition suggested by the same study is that co-expression of TIGIT in Jurkat cells affects CD226 accumulation at the interface between Jurkat and PVR-expressing Raji cells by impeding CD226 binding to PVR^10^. However, a recent study presented contradictory findings regarding the localization of CD226 and TIGIT in TCR-rich nanoclusters. Two-color direct stochastic optical reconstruction microscopy (dSTORM) analysis of activated primary human T cells with PLBs containing ICAM-1, OKT3 (anti-CD3) and either nectin-1 or PVR revealed that PVR ligation induces co-localization of CD226 and TIGIT in the cSMAC. Moreover, comparable TIGIT clustering was observed between TIGIT + CD226- and TIGIT + CD226 + T cells in the presence of PVR, indicating that CD226 binding to PVR does not interrupt TIGIT clustering at the IS when PVR is abundant (Fig. 2).

This discrepancy might result from the over-representation of TIGIT in Jurkat cells due to TIGIT over-expression. Furthermore, unlike PLBs, Raji cells endogenously express other adhesion molecules, which could indirectly influence the interaction of PVR with CD226 or TIGIT. Considering that the varied expression levels of CD226 and TIGIT depend on T-cell status, it is important to account for the different molecular stoichiometries between these molecules to understand how TIGIT directly regulates CD226 extracellularly.

##### Intracellular regulation

TIGIT has an ITIM and an ITT-like motif in its cytoplasmic domain, which exert an inhibitory signal by recruiting SH2-containing inositol phosphate-1 (SHP-1) upon ligation with PVR in NK cells^58,61,62^. Unlike in NK cells, it is still controversial whether the ITIM and ITT-like motifs of TIGIT are required for conferring inhibitory functions and CD226 inhibition in T cells.

One study with an antibody that specifically recognizes phosphorylated CD226 at Y322 (pY322) demonstrated that co-incubation of Jurkat cells expressing TIGIT WT with SEE-loaded Raji cells expressing PVR attenuated CD226 phosphorylation; this effect was not detected in Jurkat cells expressing a TIGIT mutant (Y225A/Y231A). Moreover, treatment with an anti-TIGIT mAb restored CD226 phosphorylation^9^. These findings suggest that the extracellular modulation of CD226 by TIGIT is integrated into the intracellular signaling of CD226, which is affected by TIGIT phosphorylation (Fig. 2).

However, Banta et al. proposed that the ICD of TIGIT is dispensable for inhibiting CD226 phosphorylation; instead, PD-1 recruits SHP-2 to suppress CD226^10^ (Fig. 2). When Jurkat cells expressing CD226, TIGIT, and/or PD-1 were stimulated with SEE-pulsed Raji cells expressing PVR and PD-L1, the co-expression of TIGIT and PD-1 elicited greater dephosphorylation of CD226 than did TIGIT or PD-1 alone. In addition, the expression of either an ICD deletion or a Y225F/Y231F mutant of TIGIT did not reverse the reduced phosphorylation of CD226 induced by the expression of TIGIT WT. Another study using a cell-free reconstitution system including ICDs of TCR signaling components, such as TCRζ, CD226, CD28, PD-1, and SHP2, but excluding TIGIT, also suggested the role of PD-1 in CD226 dephosphorylation. Given that PD-1 is known to localize at the cSMAC of the IS^45,63^, CD226 could also be regulated by PD-1-recruited SHP-2, which inhibits CD28 activation. However, the proposed mechanism that limits the role of the TIGIT ICD contradicts the findings of previous studies demonstrating the intracellular inhibitory function of TIGIT. Moreover, a study by Banta et al. revealed a slight increase in CD226 phosphorylation upon anti-TIGIT mAb treatment alone, which could not be attributed solely to the dissociation of CD226 from PVR without considering intracellular events. Indeed, a recent study employing various TIGIT mutants that affect glycosylation, dimerization, ligand binding, and downstream signaling further elucidated the role of the TIGIT ECD and ICD in regulating T-cell activation^55^. TIGIT mutants incapable of binding PVR fail to initiate inhibitory signals, indicating the pivotal role of extracellular events in mediating TIGIT phosphorylation-induced inhibition of T-cell responses. While this study proposed a T-cell intrinsic inhibitory role of TIGIT, irrespective of CD226, on the basis of the unaltered TIGIT clustering in the presence of CD226, it is insufficient to support a CD226-independent role of TIGIT. This finding simply implies that CD226 and TIGIT do not affect each other during the PVR ligation-induced clustering step in the overall process of regulating T-cell activation, which likely occurs in three sequential steps: (1) binding to PVR, (2) clustering at the IS, and (3) phosphorylation-mediated signal transduction.

Under physiological cell-to-cell regulation conditions, various events occur simultaneously, unlike direct regulation between molecules. Thus, discrepancies can arise from this complexity. Nevertheless, understanding how TIGIT directly regulates CD226 is crucial for designing effective anti-TIGIT therapy strategies. In particular, if TIGIT and PD-1 jointly regulate CD226 activity, this is a compelling rationale for a combined blocking strategy targeting both the PD-(L)1 and TIGIT pathways. Conversely, if TIGIT independently regulates CD226, simultaneous blockade with PD-1 may be unnecessary. Therefore, elucidating the interplay between these molecules is essential for the future clinical implementation of anti-TIGIT therapy.

##### Further considerations

Several unanswered questions remain regarding the regulation of CD226. First, there is variable co-expression of CD226, TIGIT, and PD-1 within T cells. Studies have shown an inverse correlation between CD226 and TIGIT or PD-1 in tumor-infiltrating CD8 + T cells (CD8+TILs). Thus, TIGIT and PD-1 are not always co-expressed regardless of CD226 expression in T cells from both healthy donors and patients with cancer^9,55,64^. This raises the question about the regulation of CD226 in CD226 + TIGIT + PD-1- T-cell populations if TIGIT only partially inhibits CD226 activation through competitive binding to PVR. In addition, it is important to determine whether NK cells, which rarely express PD-1^65^, employ a distinct regulatory mechanism for CD226 compared with T cells.

More importantly, it remains unclear whether PD-1 binding to PD-L1 alone can trigger CD226 inhibition or is a sequential event following TIGIT-PVR binding-mediated CD226 regulation. This is particularly important for combination therapy targeting the PD-(L)1 and TIGIT pathways, as the co-expression of PD-L1 and PVR by tumors might be necessary for this sequential event. However, a previous study reported no correlation between PVR and PD-L1 expression in five different cohorts of patients with lung adenocarcinoma^38^. Finally, the roles of other TIGIT family members, especially those related to CD226 regulation, remain elusive. While it remains controversial whether CD96 has costimulatory or inhibitory activity, CD112R is known as a co-inhibitory receptor that binds to nectin-2^33^. Therefore, understanding whether CD112R also requires PD-1 to inhibit CD226 and how it interacts with TIGIT is necessary to elucidate the broader regulatory network involving CD226.

### CD226 in tumor immunity

#### CD8 + T-cell regulation

The role of CD226 in modulating CD8 + T-cell-mediated antitumor responses has been assessed in various mouse tumor models. CD226-deficient mice presented increased tumor development and mortality after transplantation of Meth A, 3-methylcholanthrene (MCA)-induced fibrosarcoma, 7,12-dimethylbenz[a]anthracene (DMBA)-induced papilloma tumor cells or CT26^10,66^. In addition, the growth of implanted MC38-OVA tumor cells was not controlled in CD226-deficient mice^57^. In an adoptive transfer model of Pmel-1-CD8 + T cells into melanoma-bearing mice, mice given CD226-deficient Pmel-1-CD8 + T cells presented a decreased survival rate compared with that of mice given WT Pmel-1-CD8 + T cells^67^. CD226 deficiency affects the antitumor efficacy of anti-PD-1 or anti-TIGIT antibodies in B16K1- or CT26-bearing mice^10,68^. This effect was also observed in mouse tumor model studies in which anti-CD226 antagonist antibodies were used to block the interaction between CD226 and PVR. While CD226 blockade alone did not alter tumor growth or mortality in various mouse tumor models, including CT26, RENCA or lung metastasis models^69–71^, co-treatment of anti-CD226 mAb with anti-TIGIT and anti-PD-L1 mAbs or anti-PD-1 and anti-GITR mAbs inhibited antitumor responses mediated by the combined treatment with those mAbs. Immune monitoring studies with TILs revealed that CD226 blockade reversed the increased infiltration and cytokine production of CD8 + T cells induced by the combination treatment^69,71^. The discrepant effects of genetic deletion of CD226 and blockade of CD226 with an antibody on tumor control should be further assessed. Previous studies reported no alterations in T-cell development or homeostasis in CD226-deficient mice under steady-state conditions^66,71^. However, a recent study revealed that CD226 deficiency impaired the positive selection process, resulting in reduced numbers of mature CD8 + T cells^72^. Furthermore, considering that CD226 is expressed on various immune cell subsets, the genetic deletion of CD226 may alter their development or function, potentially contributing to the dysregulated tumor control observed in CD226-deficient mice. To gain deeper insight into the role of CD226 in tumor immunity, CD226 in peripheral T cells, especially within the tumor microenvironment, must be directly activated via antibody-based agonism.

Consistent with in vivo preclinical studies, in vitro evidence also suggests the importance of the CD226-PVR axis in CD8 + T-cell cytotoxicity against tumor cells. Primed CD226-deficient CD8 + T cells elicited a reduced cytotoxic effect on PVR-expressing mouse tumor cell lines, whereas CD226 deficiency did not affect the expansion of CD8 + T cells upon co-culture with mouse leukemia RMA cells that did not express PVR^66^. CD226-deficient OT-I CD8 + T cells exhibited defective proliferation upon antigen-specific stimulation with ovalbumin (OVA) peptide-pulsed EL4 cells expressing PVR^57^.

##### The impact of CD226 downregulation on CD8 + T-cell responses

Similar to T-cell adhesion molecules, CD226 is also constitutively expressed on both naïve and memory T cells at varying expression levels^9,68^. There are three different CD226 expression levels (CD226^hi^, CD226^int^, and CD226^lo^) in the memory subset, and naïve T cells harbor CD226^int^ and CD226^lo^ populations under steady-state conditions. CD226^lo^CD8 + T cells within tumors are more abundant than those in normal tissues or peripheral blood in patients with non-small cell lung cancer (NSCLC), renal cell carcinoma (RCC), multiple myeloma (MM), colorectal cancer (CRC) and CRC liver metastases^9,68,73^. Compared with CD226^hi^CD8 + TILs, CD226^lo^CD8 + TILs show reduced cytokine production and proliferative capacity^9,68,73^. Similar functional defects in CD8+TILs with CD226 downregulation have also been observed in various mouse tumor models^9,67,68,74^. Furthermore, downregulation of CD226 is often accompanied by upregulation of coinhibitory immune checkpoint receptors such as TIGIT, PD-1, Tim-3, and LAG-3 in CD8+TILs from patients with RCC, CRC, NSCLC, squamous cell carcinoma (SCC), and melanoma, which can be considered phenotypic characteristics of exhausted T cells^9,55,64^. However, some reports have shown no inverse correlation between CD226 and coinhibitory receptor expression in CD8 + TILs^67,68,73^. This disparity may be due to the heterogeneity of the population represented by CD226 downregulation or the varied status of T-cell differentiation depending on the tumor burden or variations in tumor types. Indeed, transcriptome analysis by single-cell RNA sequencing (scRNA-seq) in CD226^lo^CD8 + TILs did not reveal a consistent definition of a specific subset, such as exhausted T cells^67^. Compared with CD226^hi^CD8 + TILs, which present enriched gene expression profiles of T-cell activation and IS formation, CD226^lo^ populations present a T-cell subset with a reduced effector program, implying that CD226 downregulation is a marker for the heterogeneous dysfunctional T-cell subset. Thus, further phenotypic and functional dissection by identifying more signature markers is needed. While the tumor environment seems to promote the accumulation of CD226^lo^CD8 + T cells, CD226 downregulation is also found in resting T cells from healthy donors. A study analyzing the gene expression profiles of resting or activated CD8 + T cells with high or low levels of CD226 expression revealed that CD226^lo^CD8+ effector memory T (Tem) cells acquire gene signatures of resting T cells, regulatory T cells (Tregs) and TGF-β signaling, which support hyporesponsive CD226^lo^CD8+Tem cells upon TCR/CD28 or antigen-specific stimulation^9,68^. Unlike those of CD8+TILs, the gene expression profiles of resting CD226^hi^ and CD226^lo^CD8+Tem cells are comparable. However, it remains unclear which intrinsic or extrinsic factors are responsible for CD226 downregulation and whether CD226^lo^ memory T cells acquire characteristics distinct from those of CD226^lo^ naïve T cells. Further investigation into the trajectory and epigenetic profiles of the CD226^lo^ subset is necessary to understand how CD226 regulates CD8 + T-cell differentiation and responses. One study suggested Eomesodermin (Eomes)-mediated regulation of CD226 expression^68^. Reduced CD226 downregulation is found in Eomes-deficient CD8 + T cells, but Eomes overexpression promotes the accumulation of CD226^lo^CD8 + T-cell subsets. However, some CD8 + T-cell subsets express both Eomes and CD226^9,68^, indicating the potential involvement of other factors in CD226 downregulation. Given the key and diverse roles of Eomes in modulating CD8 + T-cell activation and differentiation^75^, it remains necessary to determine whether Eomes directly promotes CD226 downregulation or whether altered immune responses by Eomes extrinsically affect CD226 expression. Degradation of CD226 via E3 ubiquitin ligase Cbl-b-mediated ubiquitination is dependent on Y319 phosphorylation of CD226 upon its engagement with PVR^67^. Transgenic mice expressing a Y319F mutation in CD226, which results in increased frequencies of CD226^hi^CD8 + TILs, demonstrate improved tumor control. These findings may contribute to the understanding of the mechanism of CD226 downregulation at the protein level within certain contexts. For example, PVR engagement-induced CD226 degradation may not be the mechanism of CD226 downregulation in naïve T cells. Furthermore, CD226 phosphorylation at Y319-mediated T-cell inhibition, which suggests a conflicting role with that described in previous studies suggesting the importance of CD226 phosphorylation in antitumor immunity and the efficacy of TIGIT or PD-L1 blockade^9,10,43,71^, needs to be further validated at the molecular level.

#### Treg cell regulation

Tregs contribute to immune homeostasis by controlling autoimmune reactions and promoting self-tolerance in tissues^76^. Several studies have suggested that CD226 plays a suppressive role in Treg function in mouse models of inflammation and autoimmune disease^77,78^. For example, conditional knockout of CD226 specifically in Tregs decreased insulitis and delayed the onset of diabetes in female NOD mice^79^. CD226 deficiency maintains Treg function during acute graft-versus-host disease (GvHD)^80^. However, a recent study employing Treg-specific CD226 knockout mice revealed that the deletion of CD226 exacerbated the severity of GvHD and inflammatory bowel disease in animal models. The impairment of inhibitory activity in CD226 knockout Tregs has been attributed to the heightened plasticity of Tregs, which causes them to adopt a Th1-like phenotype and consequently lose their ability to suppress inflammation^81,82^. The uncertainty surrounding CD226-mediated Treg regulation may arise from variations in the expression of CD226 and TIGIT under different circumstances, as well as their functional interactions. TIGIT is highly expressed on Tregs and contributes to their immune-suppressive function^83,84^. TIGIT has been shown to restrict the PI3K-AKT pathway, thereby impeding the acquisition of a Th1 cell-like phenotype^83^. In inflammatory environments, CD226 competes with TIGIT for binding to the PVR ligand^80^. Conversely, within the tumor microenvironment, Tregs exhibit reduced expression of CD226^85^. Particularly in melanoma, human Tregs display elevated TIGIT expression and decreased CD226 expression. This leads to increased TIGIT signaling, which suppresses the PI3K-AKT pathway and enhances the suppressive functions of Tregs. Human Tregs display a considerable degree of heterogeneity^86^. Compared with Foxp3-negative CD4 + T cells, human Foxp3+ Tregs have lower CD226 levels. TIGIT expression is notably high on Tregs and increases upon activation and in vitro expansion, which is linked to lineage stability and suppressive capacity. Conversely, Tregs lacking TIGIT but expressing CD226 exhibit reduced purity and suppressive function after expansion, alongside increased IL-10 and effector cytokine production^87^. These findings suggest that the regulatory effects of TIGIT and CD226 differ between Tregs and conventional CD4 + T cells. It is essential to further determine how an imbalance in CD226/TIGIT signaling regulates the function and stability of Tregs in certain disease contexts.

### CD226 as a biomarker for cancer treatment

Emerging evidence suggests that CD226 expression is a critical determinant of the functionality of CD8+TILs and that diminished CD226 expression is implicated in conferring resistance to cancer immunotherapy. In patients with melanoma treated with ICIs such as anti-PD-1 and/or anti-CTLA-4, better progression-free survival was observed in individuals with increased frequencies of CD226 + CD8+TILs. Interestingly, the observed survival benefit was unaffected by high CD8 + T-cell infiltration^67^. In addition, CD226 expression is associated with improved clinical outcomes in patients with NSCLC treated with anti-PD-L1 therapy. In the cohorts investigated, CD28, ICOS, OX-40, 4-1BB and GITR levels within tumor tissues were not associated with better clinical outcomes^10^. When CD226 in TILs derived from CRC liver metastases was examined, patients with CD226^hi^CD8 + T cells demonstrated better survival and lower relapse rates than those with CD226^lo^CD8 + T cells. Notably, a correlation between PVR and CD226 expression in CRC with liver metastasis was not observed, suggesting other causes for decreased CD226 expression^73^. In addition, the clinical associations and prognostic value of CD226 + CD8+TILs have also been demonstrated in human gastric cancer^88^. In patients with pancreatic ductal adenocarcinoma, mFOLFIRINOX chemotherapy increases CD226 expression on peripheral blood CD8 + T cells, which is positively correlated with antigen-specific CD8 + T-cell responses following TIGIT or PD-1 blockade. Furthermore, CD226 downregulation is observed in Treg or γδ T cells in patients with cancer. In metastatic melanoma, a high TIGIT/CD226 ratio in tumor Tregs is linked to increased CD25^hi^Foxp3+Treg cell frequencies and unfavorable clinical responses following ICI therapies^85^. Elevated TIGIT + CD226-γδ T-cell levels correlate with poorer overall survival in patients with AML. Chemotherapy-induced complete remission in these patients is associated with decreased TIGIT and increased CD226 expression on γδ T cells^89^.

## CD2

### CD2 expression, structure, and ligands

CD2, alternatively referred to as LFA-2, T11 or the SRBC receptor, is a type I transmembrane glycoprotein that belongs to the immunoglobulin superfamily (IgSF)^7,90^. CD2 expression is observed on all T lineage cells, NK cells, thymocytes, and a small subset of plasmacytoid DCs (pDCs)^91,92^. Notably, while murine B cells display widespread CD2 expression, only a minority of human B cells express CD2^93,94^. The CD2 protein is composed of two ECDs, a transmembrane domain, and a cytoplasmic tail. Its ECD comprises a membrane-distal V-set IgSF domain (domain 1, D1) and a membrane-proximal C2-set IgSF domain (domain 2, D2). The core structures of the IgSF domains in rat and human CD2 closely resemble those of other IgSF domains. The adhesion function of the molecule is facilitated by the D1 domain, which binds to the corresponding ligand. In addition, CD2 clustering upon T-cell activation might involve the D2 domain^95^. The utilization of a monoclonal antibody (anti-T11_3_) capable of detecting a neoepitope named CD2R (CD2-restricted epitope) has provided valuable insights into the structural alterations occurring within the CD2 ectodomain during the activation of T and NK cells^96^. This epitope resides within the region encompassing the CD2 D1-D2 linker segment. A ligand-induced conformational change in CD2 is likely to increase the angle between the D1 and D2 domains, thereby exposing the binding site for the anti-T11_3_ antibody. This conformational change facilitates the clustering of CD2 molecules and is associated with CD2-mediated activation events^95,97^.

In humans, CD58, also known as lymphocyte function-associated antigen 3 (LFA3), is the primary binding partner for CD2. The interaction between CD2 and CD58 is characterized by a relatively low affinity (K_d_ = 9 ~ 22 μM) and rapid dissociation rate^98^. CD2 can also bind to CD48 and CD59, although with much lower affinity^99^. This finding suggests that the physiological significance of CD2 binding with CD48 or CD59 appears to be minimal in humans in vivo^100^. In mice, the CD58 gene remains unidentified, with CD48 identified as the sole counterpart for CD2. CD48 is considered the mouse counterpart of human CD58 because of its high structural and distributional similarity. CD48 interacts with both CD244 and CD2, with a preference for CD244. The affinity between hCD2 and hCD58 is approximately 50 times greater than that between mCD2 and mCD48. Furthermore, mCD2 does not interact with hCD58^90,101^. The interaction between CD2 on T cells and CD58 on APCs strengthens the adhesion between these cell types^102^. The weak binding affinity of CD2 for CD58 may facilitate rapid receptor exchange during intercellular recognition^103,104^. This heterotypic cell adhesion promotes initial cell-to-cell contact before specific antigen recognition and facilitates TCR activation by promoting interaction with pMHCs. The interaction between CD2 and CD58 in humans, or CD48 in rodents, also promotes the establishment of an optimal intercellular membrane spacing (~13 nm) between cells^97^. This distance is similar to that of the TCR-pMHC complex, facilitating the interaction of the TCR with pMHCs and potentially excluding large membrane molecules (e.g., CD45) from the center of the IS^105–107^. Overall, the presence of the hCD2–hCD58 interaction significantly enhances T-cell efficiency in recognizing the correct pMHC. Furthermore, upon cellular activation, both the CD2/CD58 affinity and LFA-1/ICAM-1 affinity increase, suggesting another means to increase cell‒cell avidity upon specific cell‒cell conjugation^102,108^. While it has traditionally been thought that CD2 primarily interacts with CD58 on APCs in *trans*, recent research has indicated that CD2 also engages in a T-cell-intrinsic *cis*-interaction with its ligands CD48 or CD58, which is necessary for enhancing TCR signaling and T-cell activation^109^. Nonetheless, the *trans*-interaction between CD2 and CD58 seems to have a greater effect on T-cell cytotoxicity^16,110^.

### CD2 in immunological synapse formation

CD2 is observed in two distinct regions of the IS. In cSMAC, it primarily serves as a costimulatory receptor, colocalizing with the CD28/CD80/CD86 and TCR-pMHC complexes. A unique protein rearrangement also occurs within the IS when sufficient CD2 molecules engage with CD58, resulting in the formation of the corolla pattern in the dSMAC. CD2 in the corolla likely engages with the cytoskeleton, stabilizing the IS. This process involves organizing signaling receptor–ligand pairs such as CD28–CD80, ICOS–ICOSL, and CD226–PVR into distinct compartments. The corolla appears to enhance the amplification of TCR signaling through the activation of PLC-γ1 and LAT^47^. The redistribution of CD2 alongside TCR/CD3 and lipid rafts in the uropod following T-cell activation suggests its involvement in prearranging the activation machinery for efficient antigen recognition^111^. The accumulation of the CD2‒CD58 complex in T-cell microvilli plays a critical role in fostering stable close-contact interactions with the glycocalyx of APCs while also assisting in the exclusion of CD45 from these sites^112^. Overall, this redistribution underscores how CD2 is instrumental in coordinating the gradual progression of T-cell activation.

### CD2 signaling

In addition to its adhesive function, CD2 plays a role in signal transduction. CD2-mediated activation depends on its ICD, which requires physical interactions with other signaling molecules to transmit the stimulus following ligand binding because of its absence of intrinsic enzymatic activity. The ICD of CD2 includes five potential domains featuring proline-rich motifs (PxxP or PxxxP), facilitating interactions with various proteins with SH3 domains^113,114^. Domains 1 and 2 bind to the Src family protein-tyrosine kinases Lck and Fyn, as well as CD2-binding protein 2 (CD2BP2)^113,115,116^. Domain 4 binds to Lck and Fyn as well as CD2BP1, CD2BP3, Cbl-interacting protein of 85 kDa (CIN85), and the Cas ligand with multiple SH3 domains (CMS, also referred to as CD2AP in rodents)^117–120^. Upon ligand-induced clustering of CD2, CD2BP1 binds to CD2 via its SH3 domain, serving as an adapter to recruit the protein tyrosine phosphatase (PTP)-PEST. This recruitment leads to the downregulation of focal adhesion and promotes T-cell motility^117^. The interaction between CD2 and CD2AP, initiated by T-cell activation, is crucial for cytoskeletal rearrangement and receptor clustering at the T-cell–APC contact region and is mediated by the first SH3 domain of CD2AP^120^. The adaptor protein CD2BP2 is capable of binding to CD2 via its GYF domain^116,121^. Fyn kinase can compete with CD2BP2 by specifically binding to the GYF domain-binding site of CD2. CD2BP2 may enhance activation signaling mediated by CD2 but not TCR/CD3-mediated signaling^113,116,121^. Nonetheless, the physiological role of CD2BP2 in CD2-mediated signaling requires further investigation. CIN85 and CD2BP3 are scaffolding proteins that drive the regulation of CD2-cytoskeletal interactions. In this model, domain 4 of CD2 binds to either CIN85 or CD2BP3 in resting cells but is degraded upon activation. This degradation allows CMS to bind to domain 4 of CD2, enabling subsequent binding to the actin cytoskeleton through the actin capping protein CapZ^122,123^. Therefore, the actin cytoskeleton can reorganize, and both the MTOC and secretory pathways can align toward the IS^124^.

The CD2–CD58 interaction enhances TCR binding to pMHC complexes, increasing TCR signaling. The TCR/CD3 complex, particularly the CD3 zeta chain, appears to be required for transmitting CD2-mediated activation signals. While CD2 and the TCR/CD3 complex have been reported to co-immunoprecipitate, direct evidence of their association is lacking^125–127^. While the engagement of CD2 by CD58 alone is insufficient for T-cell activation, their interaction can still initiate signaling by promoting the formation of CD2 clusters in distinct membrane microdomains, independent of TCR stimulation^46,128^. CD2 cross-linking can trigger T-cell proliferation and cytokine secretion, both of which are processes mediated by ZAP70 activation^129,130^. LAT is also known to be important for regulating CD2-mediated T-cell activation^129^. Both Lck and Fyn are activated following CD2 cross-linking^115,131^, and Fyn activation is linked to the activation of the PLC-γ1/Vav1/PKC/Dok/FAK/Pyk2/JNK1 axis^132,133^. CD2 signaling pathways were examined in human CD57 + CD8 + T cells activated with anti-CD2 antibodies via phosphoproteomic analysis. This revealed a broad CD2 signaling network in CD8 + T cells that significantly overlaps with the TCR-controlled network. In addition, CD2 engagement activated signaling pathways crucial for immune synapse assembly, including vesicular trafficking and cytoskeletal organization. This unique CD2 signaling pathway also influences the phosphorylation of proteins involved in immune synapse polarization. Moreover, CD2-mediated AMPK activation promotes lytic granule polarization, which contributes to the cytotoxic activity of CD8 + T cells^134^.

### CD2 in T-cell regulation

Studies with CD2-deficient TCR transgenic mice have revealed that CD2 deficiency in peripheral T cells results in reduced activation, proliferative capacity, and IFN-γ production upon antigen stimulation^135–137^. During priming, CD2 expression by naïve T cells enables reduced sensitivity to cognate antigen recognition, which allows T-cell stimulation by weak TCR agonist peptides^137^. The role of CD2 in naïve T-cell priming has also been studied using anti-CD2 antibodies^138,139^. CD2 downregulation on mouse T cells by treatment with an anti-CD2 antibody (12.15 A) led to impaired T-cell responses during priming, whereas administration of the antibody after priming had no effect on the development of antigen-specific T cells^138^. This antibody-mediated reduction in CD2 also contributes to decreased apoptosis of superantigen-responsive T cells without affecting tolerance induction by modulating the intensity of the TCR signal^139^. However, how this antibody induces CD2 downregulation and whether it blocks the interaction between CD2 and CD48 remain unclear.

CD2 costimulation is required to prevent or reverse dysfunctional states of CD8 + T cells, such as senescence, anergy, or exhaustion. The CD28-CD8 + T-cell population that is more common in elderly or chronically infected individuals^140^ is functionally defective and less proliferative^141^. CD2 is constitutively expressed in CD28-CD8 + T cells, which mostly include both EM and CD45RA+ effector memory (EMRA) subsets^142,143^. Compared with ligation with the 4-1BB ligand (4-1BBL), engagement with CD58 specifically improved the proliferation, polyfunctionality and cytotoxic activity of CD28-CD8 + T cells. In addition, CD2-CD58 costimulation restores the responsiveness of anergic T cells induced by blocking the B7 family of costimulatory molecules to alloantigens after exposure to IL-2^144^. The role of CD2 in T-cell exhaustion has been suggested in the transcriptome analysis of patients with multiple autoimmune and infectious diseases. CD2 was identified via CD4 + T-cell network analysis, which demonstrated the importance of CD4 costimulation in preventing CD8 + T-cell exhaustion^145^. Treatment with anti-CD2 agonistic antibodies but not other stimuli, such as IFN-α or anti-CD40 antibodies, allows CD8 + T cells to retain CD127 expression and viability upon persistent TCR stimulation. This ‘non-exhausted’ phenotype of CD8 + T cells was further confirmed by transcriptome analysis, which revealed that CD2 signaling limits the development of transcriptional changes associated with exhaustion.

While one previous study reported that CD2 promotes Th cell differentiation by reducing the threshold to cognate peptide rather than directly regulating Th1 or Th2 differentiation^137^, a recent finding suggested that CD58 expression by human keratinocytes causes naïve T cells to differentiate into Th1 and Th17 cells via CD2 engagement^146^. Since CD80/CD86 is not expressed on activated keratinocytes and CD28 on T cells in the epidermis of psoriatic lesions, the CD2‒CD58 axis may play an alternative costimulatory role instead of the CD2‒B7 pathway in certain conditions or locations.

The effect of CD2-CD58 costimulation on Treg responses is poorly understood. Studies with anti-CD2 monoclonal antibodies, including siplizumab and BTI-322, in non-human primates have shown that naïve T-cell and Treg depletion by anti-CD2 antibodies is limited due to low CD2 expression in these populations^147,148^. During in vitro HLA-mismatched allogeneic mixed lymphocyte reactions, proliferating CD45RA-Foxp3^hi^ Tregs were enriched in the presence of siplizumab^147^, but it is unclear how siplizumab promotes Treg expansion and whether it has intrinsic Treg cell activity or regulates it in an extrinsic way.

### CD2 in tumor immunity

Despite the suggested role of the CD2‒CD58 axis in preventing T-cell dysfunction, how CD2 costimulation regulates tumor immunity remains unknown. Several gene expression and immune monitoring studies conducted on clinical samples have reported that increased CD2 expression is correlated with improved survival rates in patients with melanoma, breast cancer, AML and diffuse large B-cell lymphoma (DLBCL)^149–152^. In addition, correlations between CD58 downregulation and resistance to cancer treatments, including ICI, CAR T-cell and R-CHOP therapies, have been reported in various malignancies, including melanoma, relapsed and refractory (R/R) large B-cell lymphoma (LBCL), DLBCL and relapsed Hodgkin lymphoma (HL)^11–16,153,154^, highlighting the nonredundant role of the CD2‒CD58 axis in antitumor immunity. Certainly, an integrative data-driven approach to identify cancer cell programs associated with T-cell exclusion has revealed that the lack of physical interaction between cancer cells and immune cells, including MHC-I-TCR and CD2-CD58, is a prominent feature of the resistance program^155^. When scRNA-seq datasets from patients with melanoma^155,156^ were further examined on the basis of the differentiation status of CD8+TILs (CD8 + TCF7+ or CD8+Tox + ) or the clinical response to ICI treatment, the majority of TILs expressed high levels of CD2 regardless of the analysis criteria^13^. Moreover, comparable levels of CD2 expression were observed in the CD19 CAR T-cell infusion products of patients with complete remission and those with progressive disease. In contrast, patients with relatively low expression of CD58 by tumor cells exhibited a significantly shorter median progression-free survival (PFS)^11^. These observations suggest that altered CD58 expression primarily contributes to immune evasion by tumor cells rather than to CD2-mediated T-cell regulation.

In vitro and preclinical studies also support the importance of the CD2‒CD58 axis in the antitumor immune response of CD8 + T cells, particularly in the context of cell‒cell conjugation. Low CD2 and CD8 expression on nonlytic TILs abrogates conjugation and subsequent IS formation with cognate target tumor cells^157^. In T-cell rosetting with HL cells, CD2 blockade or CD58 knockout inhibits rosette formation and the subsequent activation of T cells^158^. The importance of CD2 as an adhesion molecule is also emphasized in CAR T-cell therapy. When comparing the antigen recognition ability of the TCR and CAR using the C9V variant (9 V) of the cancer testis peptide antigen recognized by the 1 G4 TCR and D52N scFv, TCRs utilizing adhesion molecules, such as CD2 or LFA-1, demonstrated greater antigen recognition^159^. Compared with conventional CARs, CAR structures incorporating CD3ε extracellular domains that bind to adhesion molecules exhibit enhanced antigen recognition. In addition, deletion of CD2 from allogeneic “universal” CD19-targeting CAR T cells (UCART19) resulted in decreased antitumor efficacy^160^. A genome-wide CRISPR library screening conducted in a coculture model involving the acute lymphoblastic leukemia cell line Nalm6 and CD19 CAR T cells revealed that CD58 loss in tumor cells affects optimal IS formation with CAR T cells, resulting in impaired CAR T-cell responses^110^. Blocking the interaction between CD2 and CD58 via mAbs also caused a similar defect in the cytotoxic activity of CD19 CAR T cells against Nalm6 or Raji cells. Analysis of the interaction dynamics between CD19 CAR T cells and Nalm6 tumor cells utilizing time-lapse imaging microscopy in nanowell grids (TIMING) revealed that CD2 contributes to the directional migration of CAR T cells. This migratory ability promotes the cytotoxic activity of CAR T cells^11^. A preliminary study described the clinical relevance of the affected CD2‒CD58 axis in patients with R/R LBCL treated with CD19 CAR T-cell therapy^14^. Approximately 20% of patients with mutations or loss of CD58 expression experienced significantly decreased PFS following treatment with commercial axicabtagene ciloleucel. Another CRISPR loss-of-function and gain-of-function screen in the human ovarian carcinoma cell line OVCAR8 to identify molecules regulating T-cell engager (TCE)-mediated T-cell killing revealed that CD58 loss in tumor cells affects T-cell effector functions, along with TCR signaling induced by TCE treatment^161^.

**Fig. 3:**
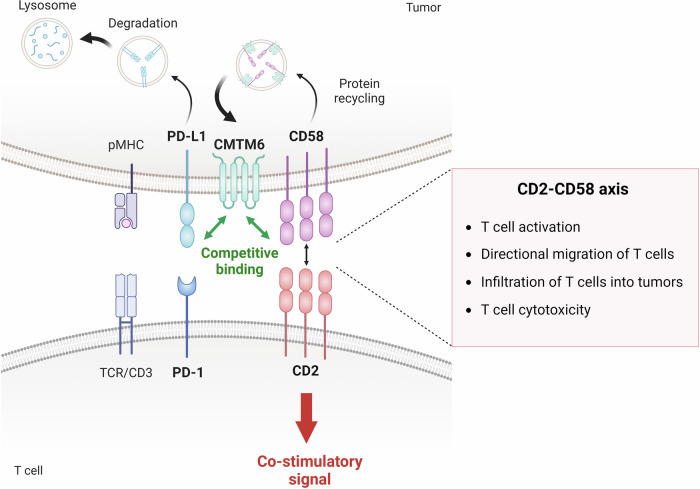
Regulation of tumor immunity and immune evasion via the CD2‒CD58 axis. CD58 downregulation on tumor cells is associated with resistance to cancer treatments such as ICI and CAR T-cell therapies. At the molecular level, this occurs when PD-L1 competes with CD58 for binding to extracellular loops within the MARVEL domain of CMTM6, leading to the downregulation of CD58 by promoting its lysosomal degradation instead of endosomal recycling. This increase in PD-L1, resulting from the loss of CD58, contributes to tumor immune evasion. In addition, impaired CD58 binding to CD2 on T cells hampers antitumor immune responses through various mechanisms, including reduced T-cell activation, directional migration, infiltration into tumors and cytotoxicity.

Recent studies have demonstrated the mechanism of CD58 downregulation in tumor cells, shedding light on the importance of CD58 as an intrinsic regulator of tumor cells (Fig. 3)^13,15^. CKLF-like MARVEL transmembrane domain containing protein 6 (CMTM6), which is known to colocalize with PD-L1 at the plasma membrane and in recycling endosomes, preventing lysosome-mediated degradation of PD-L1^162^, also controls CD58 expression via a pathway similar to that of PD-L1. Since CD58 and PD-L1 compete for binding to extracellular loops in the MARVEL domain of CMTM6, CD58 deficiency indirectly promotes PD-L1 expression via increased binding of PD-L1 to CMTM6. While CMTM6-deficient tumor cells exhibit PD-L1-dominant effects on T-cell activation, ectopic overexpression of CD58 reverses the affected T-cell activation and response to PD-L1 inhibition^162^. Interestingly, concurrent loss of CD80/CD86 expression is found in CD58-deficient tumor cells^161^. Considering the *cis*-regulation between PD-L1 and CD80/CD86, tumor-intrinsic mechanisms might coregulate the expression of CD58, PD-L1, and CD80/CD86.

In addition to the role of CD2 as an adhesion molecule, the costimulatory activity of CD2 is also required to enhance T-cell responses against tumor cells. In an in vitro model in which MM cluster entry of T cells was measured, CD2 agonism with an anti-CD2 antibody increased T-cell entry into MM clusters compared with stimulation with α-CD3/CD28 antibodies alone^163^. When CD2 binding to CD58 was blocked by treatment with an anti-CD58 antibody, the entry of preactivated T cells into MM clusters decreased in the presence of the anti-CD2 agonistic antibody, suggesting that T-cell infiltration is regulated by both the intensity of the costimulatory signal and the interaction between CD2 and CD58. Consistent with these findings, compared with CD3 stimulation alone, treatment with the CD58-Fc chimera enhances tumor cell killing by TILs. However, CD2 costimulation by binding to the CD58-Fc chimera cannot fully restore the reduced cytotoxic activity of TILs against CD58-deficient tumor cells^13^, implying that CD2-CD58 axis-mediated conjugation between T cells and tumor cells plays a major role in regulating T-cell cytotoxicity.

### Therapeutic interventions targeting CD2

Efforts to exploit the costimulatory activity of CD2 in cancer immunotherapy were initiated with the concept of a bispecific antibody (BsAb) that targets both CD2 and the tumor antigen epidermal growth factor receptor (EGFR). The mAb M2, which binds to CD2 on resting human T cells, was conjugated with an anti-EGFR mAb, resulting in the M2XEGFR BsAb. This BsAb promotes the EGFR-specific killing of A431 tumor cells by priming cytotoxic CD8 + T-cell clone C3F2 cells^164^. Treatment with M2xEGFR or the M1 clone (another mAb targeting CD2) alone fails to activate resting peripheral blood mononuclear cells (PBMCs), but combination treatment with these two antibodies induces PBMC proliferation and subsequent target cell lysis. A similar approach was undertaken by combining MUC1xCD3, MUC1xCD2 and MUC1xCD2 BsAbs^165^. PBMCs stimulated with the triple combination of BsAbs exhibited the highest cytotoxic activity against TFK-1 cells both in vitro and in vivo. Recent advances in antibody engineering technology have led to the development and evaluation of T-cell engager antibodies with tri-specificity for CD3, CD2, and tumor antigens at the preclinical level. Compared with CD3 TCE, EVOLVE-104 and EVOLVE-106, which target ULBP2 and B7-H4, respectively, confer improved antitumor responses in both in vitro and in vivo mouse tumor models^166,167^. Combination therapies utilizing costimulatory receptors such as CD28^168,169^ have recently been actively developed to overcome the limitations of conventional CD3 TCEs^170^. However, as demonstrated in recent clinical trials, such as REGN5678 (PSMAxCD28 BsAb), where two patients died^171^, CD28 agonism can lead to significant side effects^172–174^. Considering the role of CD2 costimulation in preventing T-cell exhaustion and reducing the sensitivity of TCRs to antigens, combining CD2 costimulation with CD3 TCEs may increase their efficacy. Furthermore, CD2 antibodies, which can induce resting T-cell activation^175^, may serve as alternatives to CD3 antibodies, which have the potential to mitigate hyperactivity-mediated toxicity. In a preliminary in vitro study comparing CARs with the 4-1BB endodomain and CD2 endodomain, the potential of CD2 as an alternative costimulatory molecule to 4-1BB was suggested^176^. Compared with the 4-1BB-based CAR, the CD2-based CAR maintained activation of the target cells but resulted in lower expression of IL-2 and IFN-γ. This reduction was associated with decreased secretion of IL-6 and IL-8 from macrophages, suggesting that costimulation of CAR T cells with the CD2 endodomain may not affect their antitumor efficacy or memory differentiation but may still reduce cytokine release syndrome.

## Conclusion

Given the limitations observed in the clinical application of current T-cell agonist therapies targeting TNFRSF costimulatory receptors, new strategies to amplify the activation of dysfunctional CD8+TILs by delivering potent costimulatory signals are needed. Although the precise reasons behind the limited clinical efficacy and toxicity of current T-cell agonist therapies are incompletely understood, the induction of T-cell dysfunction or deletion due to strong stimulation of exhausted T cells that have already experienced prolonged antigen exposure may contribute to the overall reduced clinical efficacy. Indeed, both preclinical and clinical studies have reported T-cell exhaustion and activation-induced cell death due to overactivation in response to concurrent treatment with anti-PD-1 and anti-OX40 or anti-4-1BB or an IDO inhibitor as well as continuous treatment with CD3 TCEs^6,177^. These findings suggest that the strength of the costimulatory signal may not be the key consideration for reinvigorating exhausted T cells. Rather, it may be more crucial to understand the T-cell status in a context-dependent manner and apply the appropriate agent accordingly. For example, recent studies have revealed that proper T-cell activation by blocking the PD-1/PD-L1 pathway at the tumor-draining lymph node plays a crucial role in systemic antitumor immunity by generating TCF1 + TOX+ progenitor exhausted T cells (_TPEXs_)^178–180^. Lowering the threshold for antigen affinity can also significantly enhance tumor antigen-specific T-cell activation. One key role of T-cell adhesion-costimulatory molecules, including CD226 and CD2, is to support antigen recognition by T cells by promoting IS formation. In particular, PD-1 accumulation compromises CD2-corolla-mediated signal amplification^47^, indicating that both CD2-CD58 costimulation and PD-1 blockade synergistically facilitate T_PEX_ generation. Moreover, altered activation or expression of both CD226 and CD2 is linked to dysfunctional or exhausted T cells, underscoring their importance in maintaining the functional activity of T cells.

Another critical requirement for bolstering the antitumor immune responses of CD8 + T cells, apart from their ability to carry costimulatory signals, is their ability to form conjugates with tumor cells. In cases of treatment-resistant tumors exhibiting low CD58 levels, the deficiency in T-cell conjugation enables immune evasion by tumor cells, regardless of intact costimulation signals. This highlights the importance of enhancing tumor cell eradication by T cells without necessitating the triggering of strong costimulatory signals.

To date, only one anti-CD226 agonist antibody, LY3435151 (Eli Lilly), has reached clinical trials (NCT04099277). Although the study was terminated early for unclear reasons, it highlighted the potential of CD226 agonists as a therapeutic avenue. While CD226 and CD2 present novel qualities as targets for T-cell agonist therapy, questions remain regarding their mechanisms of action and strategies for exploitation. Both molecules are constitutively expressed from naïve T cells to memory and effector T cells. Although their expression levels are greater in memory and effector T cells than in naïve T cells, stimulating naïve T cells in lymphoid organs rather than tumor sites may increase the risk of immune-related adverse events. Selecting the Fc portion for agonistic effects may also lead to lymphocyte depletion, which requires strategies such as tumor-targeting BsAbs.

Understanding the causes and implications of CD226 downregulation in CD8+TILs is crucial for developing effective therapies. Thus, clarifying how TIGIT and/or PD-1 regulate CD226 activity is essential because conflicting findings are needed to determine whether patients with high PD-L1 or high PVR should be targeted for CD226 agonist therapy.

The structural changes induced by T-cell activation are important for CD2 antibody epitope selection to initiate proper CD2 costimulation. Moreover, CD2 can regulate its function through both *trans*- and *cis*-interactions with CD58, which should be considered in the development of CD2 agonistic antibodies. Previous studies have shown that, depending on the epitope, CD2 alone can activate T cells independently of TCR stimulation^175^, highlighting the need to understand the characteristics of T cells stimulated by CD2 as the primary signal. While the intrinsic costimulatory signal of CD2 in T cells remains important, leveraging its interaction with CD58 on tumors appears to be a pivotal mechanism in the regulation of tumor immunity and requires novel strategies.
